# Challenges in DICER1-Associated Lung Disease

**DOI:** 10.3390/jcm12051918

**Published:** 2023-02-28

**Authors:** Kamal Masarweh, Oz Mordechai, Michal Gur, Ronen Bar-Yoseph, Lea Bentur, Anat Ilivitzki

**Affiliations:** 1 Pediatric Pulmonary Institute, Ruth Rappaport Children’s Hospital, Rambam Health Care Campus, Haifa 3109601, Israel; 2 Pediatric Hematology and Oncology Department, Ruth Rappaport Children’s Hospital, Rambam Health Care Campus, Haifa 3109601, Israel; 3 Rappaport Faculty of Medicine, Technion–Israel Institute of Technology, Haifa 3200003, Israel; 4 Radiology Department, Rambam Health Care Campus, Haifa 3109601, Israel

**Keywords:** pleuropulmonary blastoma, DICER1, lung cyst

## Abstract

Pleuropulmonary blastoma (PPB) is a tumor occurring almost exclusively in infants and young children. This is the most common primary-lung malignancy in childhood. There is age-associated progression through a distinctive sequence of pathologic changes, from a purely multicystic lesion type I to a high-grade sarcoma type II and III. While complete resection is the cornerstone treatment for type I PPB, aggressive chemotherapy with a less favorable prognosis is associated with type II and III. DICER1 germline mutation is positive in 70% of children with PPB. Diagnosis is challenging, as it resembles congenital pulmonary airway malformation (CPAM) in imaging. Although PPB is an extremely rare malignancy, over the past five years we have encountered several children diagnosed with PPB in our medical center. Herein, we present some of these children and discuss diagnostic, ethical, and therapeutic challenges.

## 1. Introduction

Pleuropulmonary blastoma (PPB) the most common primary lung malignancy in childhood [1]. First recognized in 1988, it is considered a dysembryonic equivalent to embryonal malignancies such as neuroblastoma, hepatoblastoma and other organ-based solid malignancies of childhood [2]. PPB is a rare tumor, occurring almost exclusively in infants and young children under 6 years of age, similar to other embryonal malignancies [3].

PPB is unique among developmental malignancies of childhood in its age-associated progression through a distinctive sequence of pathologic changes from a purely multicystic lesion to a high-grade sarcoma. Type I PPB is composed of air-filled cysts with primitive mesenchymal cells beneath an intact, benign-appearing epithelium. These cysts can progress into type II PPB, in which the mesenchymal cells overgrow the septa, producing a cystic and solid neoplasm. Type III PPB is an exclusively solid sarcomatous neoplasm [2,3].

There is strong evidence for progression from type I to type II and type III PPB. Type Ir (regressed) PPB is a purely cystic lesion, microscopically devoid of subepithelial septal primitive cells, and it is considered to have a lower risk for neoplastic progression [4,5]. The pathological progression in the PPB types correlates with both age at diagnosis and clinical outcome [5]. Type I and type Ir present at a younger age, and the prognosis is favorable, with a 5-year survival of 98% and 100% of patients, respectively. Type II and type III PPB are typically diagnosed in older children, with 5-year-overall-survival estimates of 75% and 53%, respectively [6].

Type I PPB may present with respiratory symptoms such as cough, dyspnea, chest pain or abdominal pain. Chest X-rays may show signs of pneumonia, and some cases may present with difficulty in breathing, from a pneumothorax or a large lung cyst, or a PPB can also present as an incidental radiologic finding of a lung cyst. The purely cystic type I PPB can be easily mistaken for other congenital lung malformations such as congenital pulmonary airway malformation (CPAM), as CPAM origins can be from the acinar structures of the lungs, thus creating a multiseptated cystic lesion similar to type I PPB. The diagnosis of type I PPB should be considered in the evaluation of any multicystic specimen of peripheral lung from young children. The final diagnosis requires meticulous pathological examination.

Given the rarity and clinical heterogeneity, it is still unclear what is the optimal therapeutic approach. The treatment for type I and type Ir PPB evolved over time; most cases are treated with surgical resection alone, while in some cases adjuvant chemotherapy is added. Most type Ir PPBs are followed clinically after complete surgical resection, due to the low risk of tumor progression. Cases of type I PPB are treated with oncologic surgery alone. In cases of suspected incomplete resection or unresectable lesion, chemotherapy is added [7]. A recent study found that adjuvant chemotherapy in cases of complete resection in type I PPB was found to be protective, with lower rate of recurrence of progression compared to surgery alone [8]. Type II and type III PPB have a more aggressive clinical behavior, requiring both surgery and chemotherapy [9].

PPB may be associated with other unique malignant syndromes. An important discovery was the identification of the pathogenic germline *DICER1* variants as the first known genetic causes of PPB familial syndrome [10], and these variants are found in 70% of patients with all types of PPB. DICER1 is inherited in an autosomal dominant condition with variable penetrance. In addition to PPB, DICER1 syndrome includes, among others, Wilms’ tumor, cystic nephroma, clear cell sarcoma, ovarian Sertoli-Leydig cell and granulosa cell tumors, testicular Sertoli-Leydig cell tumors, medulloblastoma, neuroblastoma, seminoma germ cell tumors, pituitary blastoma, pineoblastoma, ciliary body medulloepithelioma, thyroid nodular hyperplasia, papillary and follicular thyroid carcinomas, rhabdomyosarcoma, fibrosarcoma, Ewing sarcoma, osteosarcoma, hepatoblastoma and hepatocellular carcinoma cancer and pineoblastoma [11]. Germline DICER1 mutations are also associated with non-neoplastic conditions, including macrocephaly, retinal abnormalities, renal anomalies, dental perturbations, and GLOW syndrome (global developmental delay, lung cysts, overgrowth and Wilms’ tumor) [12]. DICER1 is an important gene, located on chromosome 14q32.13 in the biogenesis of microRNAs, a class of small RNA molecules essential in organ development and suppression of neoplasia [13]. Deficient microRNA suppression can lead to oncogenic transformation [14]. In contrast to loss-of-function germline *DICER1* mutations, missense mutations in the RNase IIIb domain of *DICER1* are found as ‘second hits’ in tumors associated with germline mutations [15].

The International PPB/DICER1 Registry (IPPBR) was founded in 1987 to advance research and improve outcomes for children with PPB and DICER1 syndrome. The registry allows free central-pathology review in questionable cases, review of records in a standardized method, longitudinal follow-up and worldwide collaboration. Since its inception, more than 800 patients from 47 countries have enrolled in the registry [9]. The registry’s research includes efforts to define optimal therapy regimens for PPB and DICER1-related cancers and to discover new therapies for DICER1-related cancers, develop new ways to diagnose children with DICER1-related cancers and present surveillance guidelines for patients with germline DICER1 mutations [16]. Based on information collected from the registry, a study by Hill et al. identified germline loss-of-function DICER1 mutations affecting the RNase IIIb domain in affected families with PPB and a spectrum of other tumors in the DICER1 syndrome [10].

Although PPB is considered a rare malignancy, several children have been diagnosed with PPB in our medical center in the last 5 years. We present some of these cases, clinical, radiological, pathology and genetic-testing findings, and discuss challenging questions that were raised regarding diagnosis, optimal management and treatment.

### 1.1. Patient No 1

**Figure 1:**
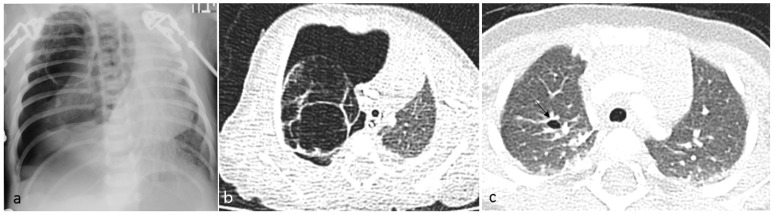
Six-day-old infant with respiratory distress. (**a**) Chest X-ray on day two of life: cystic lesion and tension pneumothorax on the right. (**b**) Chest CT without IV contrast performed on the same day, axial image: multicystic lesion and adjacent pneumothorax on the right hemithorax. (**c**) Chest CT performed 9 months later, with tiny cystic lesion on the right (arrow).

**Figure 2:**
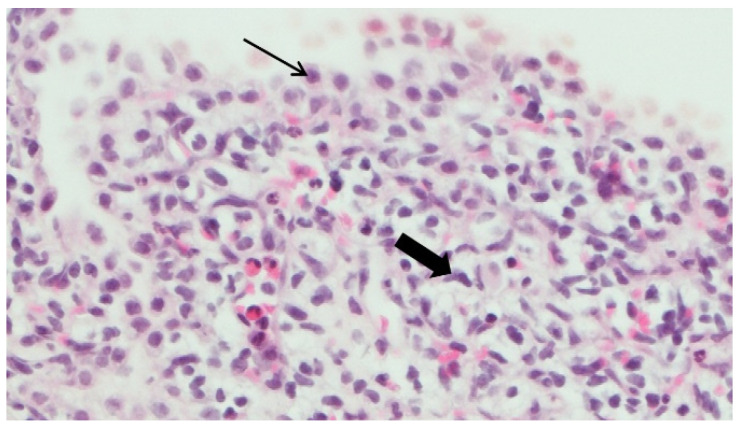
Pathology specimen of the cystic lung lesion in case number 1 (H&E). Alveolar cell (thin black arrow) and spindle cell (thick black arrow).

A 6-day-old term girl presented with severe respiratory distress and a large pneumothorax on the right side, requiring chest drainage and a prolonged course of treatment, including intubation (Figure 1a). Chest computed tomography (CT) showed a pneumothorax and a cystic mass on the right upper lobe (Figure 1b). Although lobectomy was recommended, she underwent segmentectomy on day 11 of life. The pathologic diagnosis was CPAM type 4. Due to the early pneumothorax and normal prenatal ultrasound, pleuropulmonary blastoma was suspected. Genetic counseling revealed *DICER1* germline mutation. We consulted the IPPBR, and they revised the pathologic specimen. Based on the cellularity and hyperchromasia of spindle cells within the cyst walls, a diagnosis of type I PPB was confirmed (Figure 2). Completion lobectomy, chemotherapy or close follow-up were suggested as treatment options. The parents chose a follow-up regimen. Nine months later, a new bullous lesion (6 mm) appeared in the right upper lobe (RUL) (Figure 1c). Consequently, a completion RUL lobectomy was performed. The patient received no chemotherapy. Currently she is under close monitoring in our pediatric pulmonology and haemato-oncology units, with the periodic evaluations of a *DICER1*-positive patient, including chest CT scans. She is now 5-year PPB-event free, and thriving.

### 1.2. Patient No 2

**Figure 3:**
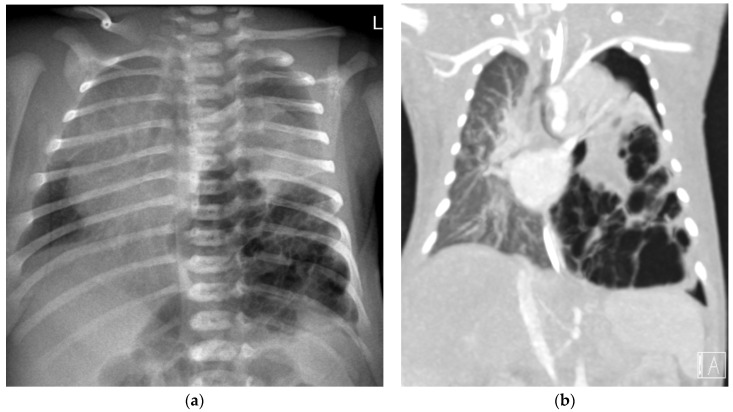
Two-day-old infant with respiratory distress. (**a**) Chest X-ray and (**b**) Coronal image of CTA: Large multilocular cystic lesion in the right lower hemithorax, with mediastinal shift to the right. There is an atelectasis of the left upper lung and apical pneumothorax.

A 2-day-old term girl presented with severe respiratory distress. Chest X-ray showed large multicystic lesion on the left hemithorax with compressive atelectasis of the lung, small pneumothorax and mediastinal shift to the right (Figure 3a). Prenatal follow-up was normal. An urgent thoracoscopy and positioning of chest tube was performed, followed by CT angiography (CTA) that better delineated the same findings, suggestive of type 1 PPB (Figure 3b). Under left thoracotomy she underwent left lower-lobe lobectomy with complete resection of the mass. The pathologic diagnosis was type 1 PPB, positive *DICER1* in the specimen (using PCR amplification with full exonic coverage, followed by NGS sequencing), but negative *DICER1* germline. More than a year later, she is well.

### 1.3. Patient No 3

**Figure 4:**
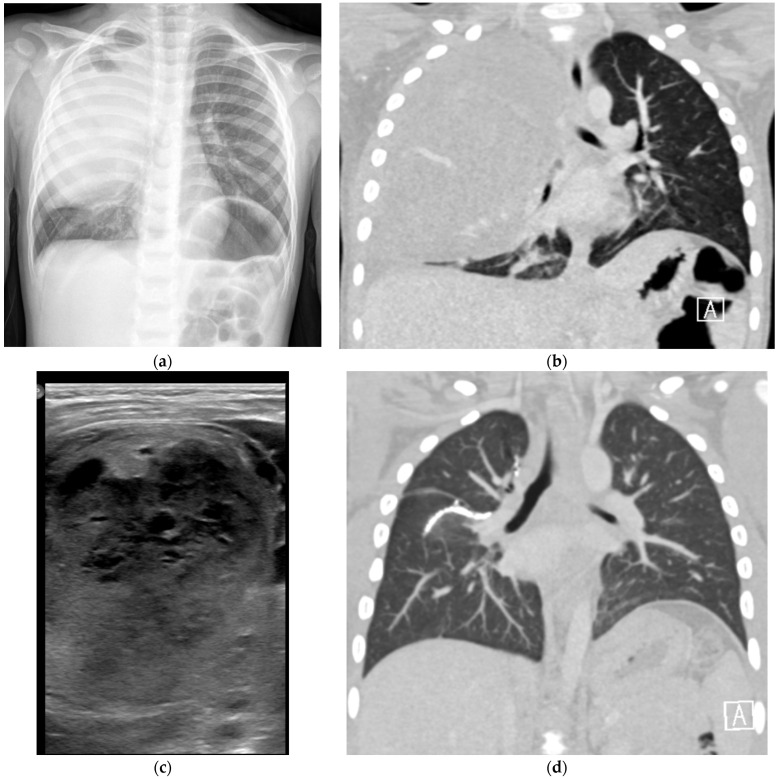
Six-year-old boy with progressive shortness of breath, fatigue and weight loss. (**a**) Chest X-ray: Opacification of the upper two thirds of the right upper hemithorax by a pleural-based lesion. There is a slight shift of the trachea to the left, and a caudal shift of the right diaphragm. (**b**) Coronal image of CTA showing large aberrant vessels within the lesion. (**c**) Sonographic gray-scale high-resolution image of the lesion (linear array transducer) shows the cystic/solid nature of the lesion. (**d**) Coronal image of CTA at the end of treatment, showing no evidence of recurrent disease.

**Figure 5:**
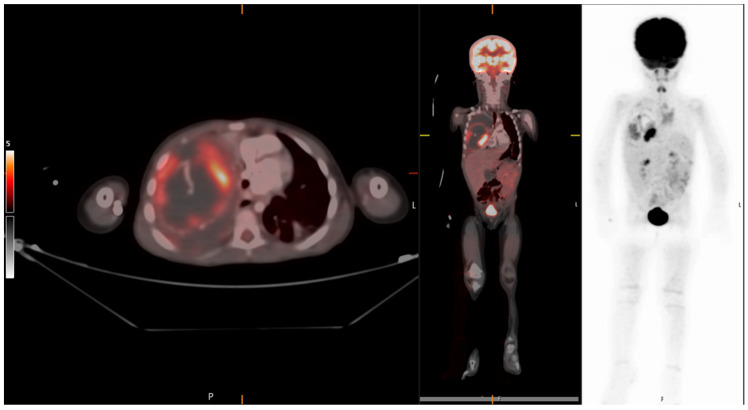
The same patient as in image 4. FDG PET-CT in the axial and coronal hybrid images and coronal PET image show peripheral uptake in the lung lesion. No metastases seen.

A 3-year-old healthy boy experienced increasing weakness and shortness of breath several weeks after a febrile upper-respiratory disease. He lost 1 kg of weight. A chest X-ray performed in the community showed complete opacification of the right hemithorax (Figure 4a), and he was referred to the hospital. A chest CTA revealed a large tumor occupying most of the right hemithorax, without any aeration of the lung (Figure 4b). Most of the tumor was solid hypodense tissue, with some cysts on the periphery. An ultrasound study was able to better delineate the multicystic hemorrhagic nature of the tumor (Figure 4c). A biopsy was taken under sonographic guidance and the pathologic diagnosis was type II PPB with foci of RMS (rhabdomyosarcoma). The *DICER1* germline was positive, and staging with FDG PET-CT (2-[(18)F]-fluoro-2-deoxy-d-glucose positron emission tomography-computed tomography showed no metastases (Figure 5). The tumor was unresectable, and the patient received neoadjuvant chemotherapy with IVADo (Ifosfamide, vincristine, actinomycin-D, and doxorubicin) followed by resection of the tumor, which was pleural based. He received completion chemotherapy of up to 12 courses. There was 80% tumor necrosis in the final specimen. After 3 years of follow up, both clinical and with CTA, there is no recurrence (Figure 4d). The *DICER1* germline mutation was positive in the patient’s father and in his three siblings. The siblings were all asymptomatic. They all underwent CTA of the chest, with the following findings:

### 1.4. Patient No 4

**Figure 6:**
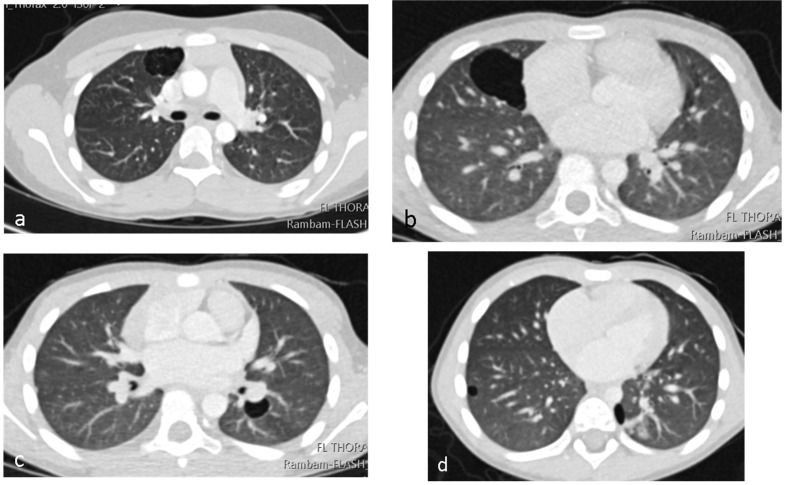
Asymptomatic *DICER1*-germline-positive siblings of index patient number 3. Axial images of Chest CTA: (**a**) Nine-year-old girl with RUL anterior multiseptated cystic lesion. (**b**,**c**) Six-year-old boy with right and left cystic lesion. (**d**) Fifteen-month-old boy with bilateral, small cystic lesions.

Nine-year-old girl (the older sister of patient no 3): complex cystic lesion with aberrant vessels within the cyst septa located in the right upper lobe (Figure 6a). We suspected that this lesion might be PPB, and consulted the International Pleuropulmonary Blastoma registry team. They agreed, and suggested lobectomy.

### 1.5. Patient No 5

Six-year-old boy (older brother of patient no 3): two purely cystic lesions, one in the right middle lobe (diameter of 4 cm) and a smaller cyst in the left lower lobe (Figure 6b,c).

### 1.6. Patient No 6

Fifteen-month-old boy (younger brother of patient no 3): two purely cystic lesions, one in the right lower lobe (diameter of 0.6 cm) and a second cyst (diameter of 1 cm) in the left lower lobe (Figure 6d).

We consulted the IPPBR team about the other three siblings as well; they considered the lesions to be a type I PPB, and resection was suggested.

The parents were given the complete information about the findings and the consultations. They decided to proceed with a more conservative approach, with clinical and radiological follow up only. Two years later, the children are healthy, and the radiological findings are stable.

## 2. Discussion

We presented six children with either proven PPB or DICER1-related cystic lung disease suspected to be PPB, all treated in our hospital within the last 5 years. This cluster of patients raised our awareness about this rare tumor and the diagnostic and management challenges it brings. Timely diagnosis and resection of type I PPB is crucial, but this entity is elusive, masquerading as a lung malformation, or growing silently in *DICER1*-germline-positive patients. We summarize some of the challenges we dealt with while treating these patients.

### 2.1. Challenge No 1

Can we differentiate CPAM from type 1 PPB, based on clinical aspects and imaging?

Differentiating the entities may influence management. There are different approaches for the management of CPAM. Some centers advocate an aggressive approach by resection of all lesions, in fear of future progression to malignant PPB. Others choose to follow up, advocating that PPB is a rare entity and CPAM 1–3 are benign lesions that will not progress to PPB [17]. The real concern is CPAM type 4 that might progress to type I PPB, so defining clues for diagnosis will allow for more judgmental choice of management.

Imaging alone cannot differentiate CPAM type 4 from type I PPB [5,17,18]. In a recent retrospective study by Engwall-Gill et al., nine pediatric radiologists reviewed the CT scans of patients with postnatal cystic lesions, and could not find any specific imaging characteristics that could safely differentiate type I PPB from congenital lung malformation. The sensitivity for diagnosis of PPB was 58%, with a specificity of 83%, with poor interrater reliability (κ = 0.36; *p* < 0.01) [19].

What are the differences between CPAM type 4 and type I PPB? Dehner et al. stated that CPAM type 4 and type I PPB are in fact synonymous, and, due to the concern of progression from type I PPB to the malignant type II and III PPB, it would be wiser to define these lesions as type I PPB rather than CPAM 4, and thus promote a more aggressive approach toward oncologic resection and genetic counselling [20].

In Feinberg A. et al., the IPPBR team summarized their experience with the goal of answering this question. Overall, 112 cases of type I PPB gathered in the registry between 2002 and 2013 and 103 cases of CPAM that were resected in the same institution during the same period were reviewed. Features favoring CPAM were prenatal diagnosis, and CTA findings of a systemic feeding vessel and hyperinflated lung. Features that raised the suspicion of PPB were multi-segmental or bilateral lesions and the absence of prenatal ultrasonography findings [18].

In our first two patients, the suspicion of type I PPB was high, due to the early symptomatic cystic lung lesion and normal prenatal follow up. In the recent IPPBR report, 85% of type I PPB cases were symptomatic at presentation, 32% with pneumothorax [18]. The positive-*DICER1* germline mutation further confirmed our concern. We consulted the IPPBR, which supported our diagnosis of type I PPB. In the family with four children with DICER1 (patients 3–6), two of the three asymptomatic siblings have bilateral cystic lesions, and thus they are highly likely to having type I PPB.

### 2.2. Challenge No 2

What are the indications for *DICER1* germline testing? Any congenital cystic lesion? Only in cases that are highly suspicious? Only in pathology-determined PPB?

In 2016, the IPPBR convened an international DICER1 symposium and published a consensus paper on the indications for testing and the surveillance advised for DICER1-positive children [16]. The paper presents recommendations for genetic testing, prenatal management and suggested signs and symptoms and imaging-surveillance strategies for DICER1-associated conditions including pulmonary, renal, gynecologic, thyroid, ophthalmologic, otolaryngologic, central nervous system tumors and gastrointestinal polyps [16]. Any clinical pathology that might be related to DICER1 was considered as a criterion. Pathology-confirmed PPB is considered a major criterion, as well as any lung cyst in childhood. Based on this consensus, any major criterion, two minor criteria (such as cystic lung disease in adulthood and renal cysts), or a first-degree relative with positive-germline DICER1 should be tested.

Recommendation for screening in *DICER1*-germline-positive children are age based, with at least one chest CT performed in all cases [16]. These indications for testing and screening elevated the awareness about PPB among pediatric caregivers, and increased the diagnosis of type I PPB and type Ir PPB.

Based on the IPPBR recommendations, we tested and diagnosed the siblings of patient number 3, who presented with malignant PPB: all three siblings had CT findings suggestive of PPB. The family decided to continue with clinical and radiological follow up instead of resecting all lesions. This family evoked an ethical question regarding the indications for testing an asymptomatic sibling. The parents of patient no 3 stated during the follow up, post factum, that they preferred not to know the child’s DICER1 status. With increased awareness of the advantages of early detection of type I PPB, and the possible prevention of progression through early intervention, more families will eventually choose to know the DICER1 status. These four DICER1 siblings bring us to the next challenge.

### 2.3. Challenge No 3

When can we safely say that a type I PPB lesion has regressed to Type Ir PPB?

We know that pathology alone can provide the final discrimination between type I and Ir PPB, but in our DICER1 family, pathology was not available for the three siblings of patient no 3. The children differ in age and appearance of the lesions. The oldest sibling was 9 years old when diagnosed, and had a multiseptated single cyst, while the two younger brothers had multifocal simple cysts. Over 2 years of CT follow up, we saw no change in the appearance of the lesions in all three children (patients Nos. 4–6). Does it give us any reassurance? We know that the median age for diagnosis of type I PPB is 7 months, for type II PPB 35 months and for type Ir PPB 2.6 years [4,5,8], and that after the age of 3 years, type Ir PPB is considerably more common than type I [8]. Were they all type Ir PPB at presentation? Can type Ir PPB progress to a malignant PPB?

The IPPBR reported two cases of progression in type Ir PPB, both occurring within 2 years. They suggested 2 years of follow up for resected type Ir PPB. In type I PPB recurrence occurred more frequently and within 63 months, so they advised a longer follow up for type I PPB, of at least 6 years [8]. We intend to continue the follow-up of the DICER1 family for at least 6 years. This family is registered in the IPPB registry, and this follow-up may shed additional light on type I PPB DICER1-related tumor.

### 2.4. Challenge No 4

Multifocal cystic lesions in a *DICER1*-germline-positive, asymptomatic child. Oncologic resection versus close radiologic and clinical follow-up: pros and cons.

Multifocal cysts are more difficult to manage. On the one hand, multifocal cysts have an increased risk of progression compared to a single cyst, while on the other hand multiple surgeries might increase surgical complications and decrease lung function. In the last IPPBR report [8], 25% of cases of type I PPB I were multifocal, and many of them had multiple surgeries at an early age. Some cysts were not resected, due to their location. Only one child had a serious complication of pulmonary artery stenosis. At the moment, the IPPRB’s recommendation in multifocal cystic PPB is to individualize management, as additional studies are needed [8].

### 2.5. Challenge No 5

The role of chemotherapy in cases of suspected incomplete resection of type 1 PPB.

PPB Ttpes II and III are aggressive malignant diseases and chemotherapy is mandatory. The primary treatment for type I and Ir PPB is surgery with complete resection. The reasoning for adjuvant chemotherapy in type IPPB and even type Ir PPB is to prevent recurrence or progression of any residual disease (microscopic or macroscopic) to the more aggressive tumor type II and type III PPB. In the recent report by the IPPBR [8], the group summarized their experience with type I PPB and type Ir PPB cases treated between 2006 and 2022. Altogether, they had 118 cases of type I PPB and 87 cases of type Ir PPB. In the PPB cases, chemotherapy (VAC, VA or IVADo) was given to 39% of the cases after surgery and in one case prior to surgery. In this study, chemotherapy proved protective, as none of the cases that received adjuvant chemotherapy progressed. For the cases that were not treated with chemotherapy, two factors were found to correlate with recurrent disease or progression: inadequate surgery, meaning less than complete resection, and multicentric disease [8]. A prior publication by the same group did not find any benefit from chemotherapy for type I PPB [6]. In patient 1, the first surgery was inadequate, and a local recurrence occurred. Following completion of the lobectomy, we consulted the IPPBR, and they suggested adding adjuvant chemotherapy. A close follow-up was decided upon, and no chemotherapy was given.

### 2.6. Challenge No 6

Is the prevalence of Type I PPB increasing in recent years, or have we misdiagnosed some of them as CPAM?

Within five years we diagnosed six patients with DICER1 due to high level of suspicion, five of them were diagnosed with type I PPB. We consulted the IPPBR team in all our cases and learnt that there is a need for a high level of suspicion and experienced teamwork in order not to miss the type I PPB patients and to be able to treat them accordingly. It is possible that the prevalence of type I PPB is on the rise, due to the fact we might have misread some of our previous cases as CPAM. In fact, to emphasize the importance of a central pathology review for rare tumors such as PPB, it was reported that almost 20% of the pathology specimens submitted for review by IPPBR had another initial diagnosis. Thus, it may be challenging to identify a lesion as PPB, even for experienced pathologists [6].

## 3. Conclusions

PPB is a rare tumor, with predicted progression from the benign type I PPB to malignant tumor. Timely diagnosis and complete resection of type I PPB correlates with the best prognosis. Symptomatic cystic lung lesion in infants, unidentified during pregnancy, especially when septated or multifocal, should raise the suspicion of type I PPB rather than CPAM, and induce testing for DICERI mutation and early complete resection. The management of multifocal cysts, the role of chemotherapy and management of older asymptomatic DICER1 children is complicated, and should sometimes be individualized. Consultation with the IPPBR team is recommended.

## Data Availability

The data presented in this study are available on request from the corresponding author.
